# Resilience in neurodivergence: professional perspectives mapped to the World Health Organisations’ International Classification of Functioning

**DOI:** 10.1038/s41598-025-25079-0

**Published:** 2025-10-27

**Authors:** Melissa H. Black, Julie Segers, Soheil Mahdi, Cecilia Ingard, Vincent Grimaldi de Puget, Sven Bölte

**Affiliations:** 1 Department of Community and Clinical Health School of Allied Health, Human Services & Sport La Trobe University, Melbourne, Australia; 2 Center of Neurodevelopmental Disorders (KIND), Department of Women’s and Children’s Health, Centre for Psychiatry Research, Karolinska Institutet & Region Stockholm, Stockholm, Sweden; 3 R2D2-MH Adult Co-Creation Group, Antwerp, Belgium; 4 Department of Philosophy, University of Antwerp, Antwerp, Belgium; 5 Parenting and Special Education Research Unit, Faculty of Psychology and Educational Sciences, KU Leuven, Leuven, Belgium; 6 CEREB, Stockholm, Sweden; 7 Faculty of Health and Occupational Studies, Department of Social Work and Criminology, University of Gävle, Gävle, Sweden; 8 Think_Differently - Paris Lab for Neurodiversity at Work, Ecole des Ponts Business School, Institut Polytechnique de Paris, Palaiseau, France; 9 Child and Adolescent Psychiatry, Stockholm Health Care Services, Region Stockholm, Stockholm, Sweden; 10 Curtin Autism Research Group, Curtin School of Allied Health, Curtin University, Perth, Australia

**Keywords:** Neurodiversity, Resilience, International classification of functioning, Disability and health, Survey, Health care, Neuroscience, Psychology, Psychology

## Abstract

Individuals with childhood-onset divergent neurological development, such as autism or Attention-Deficit Hyperactivity Disorder (ADHD), may live a good life according to objectively or subjectively determined standards. Yet, most research has focused on deficits and risks for negative outcomes. This international study forms part of a larger project examining the factors contributing to well-being, mental health, and functioning outcomes in neurodivergent populations using the World Health Organization (WHO) International Classification of Functioning (ICF). Following ICF research branch methodology, 198 professionals were surveyed on the factors that they believe are important for risk and resilience in neurodivergent populations and linked responses to ICF nomenclature using a standardized linking process. A range of bio-psycho-social factors perceived to be important for risk and resilience in neurodivergent populations were identified, including temperament and personality, emotional functions, the structure of the brain, financial status, recreation and leisure, and the immediate family. Most factors identified were environmental or related to activity and participation. Findings add to the limited literature on resilience in neurodivergent populations. We identify risk- and resilience-inducing factors that operate in the context of neurodivergence across the lifespan. These serve as candidates for future investigation and provide targets for intervention and social participation support.

**Supplementary Information:**

The online version contains supplementary material available at 10.1038/s41598-025-25079-0.

## Introduction

Individuals with altered inborn or childhood-onset neurological maturation or functioning represent a significant minority of the population^1^. For the purposes of discussion, we adopt the term “neurodivergent” to refer to this diverse group, which includes individuals with neurodevelopmental conditions such as autism and Attention-Deficit Hyperactivity Disorder (ADHD), who demonstrate divergent neurological functioning that influences their perception and interaction with the world around them^2^. At a population level, these groups experience poorer outcomes compared to the general population in diverse areas such as education, employment, and mental health^3–6^.

While population-level statistics can paint a bleak picture of neurodivergence, even population studies show that there is considerable heterogeneity in functional and well-being outcomes, and many neurodivergent individuals can achieve normative standards for positive life outcomes^3–5^, or experience a good life according to other subjectively determined criteria^7^. Despite many neurodivergent individuals attaining positive life outcomes, little research has examined the factors that can support the achievement of these outcomes. Current research and practice remain largely rooted in a risk and deficit approach. This over-reliance on negative factors places disproportionate emphasis on individual challenges as the causes of disability alone, neglecting that environmental factors likely play a significant role^8,9^. This framing also often fails to capture that neurodivergent people might also present with resources, strengths, or enablers, which could help to explain heterogeneity in neurodivergence and foster agency. It also overlooks many factors that may promote more positive outcomes^10^. There is thus a need to challenge and expand beyond conventional risk-focused views of neurodivergence.

With this view, resilience may provide an important avenue for investigation. Rather than focusing on risk and deficit, resilience is concerned with the notion of positive adaptation despite adversity^11,12^. Thus, exploring resilience shifts focus away from the factors that contribute to poor life outcomes, to instead examining what may promote positive outcomes and protect against negative trajectories. While resilience can be understood as a trait, more contemporary approaches to resilience tend to frame it as a dynamic process of positive adaptation in the face of adversity, comprising the interaction of multi-system protective and promotive factors^12^. Process-based definitions of resilience mean that bio-psycho-social factors contribute to resilience and suggest that it may be modifiable if influential factors are identified and harnessed^12^. In populations where resilience has received greater attention, such as mental health, research has shown that it involves complex interactions between a range of protective and promotive factors, including internal (biological and psychological systems) and external (environmental and sociological) mechanisms^12^. Further complicating the matter, evidence suggests that the operation of these factors seems to be moderated and influenced by contextual variables such as culture and gender^12,13^. In light of the complexities associated with the manifestation of resilience, it is likely that neurodivergence may also influence the operation of resilience-inducing factors, thereby influencing the extent to which these factors protect against adversity or promote positive outcomes.

Unfortunately, very little research to date has examined resilience specifically in neurodivergent populations, especially using bio-psycho-social, process-based conceptualizations^14^. Existing research in different neurodivergent populations, such as those with learning disabilities^15^, autism^16,17^, and ADHD^18^, suggest that there may be similar bio-psycho-social influences on resilience. Still, there are also indications that factors impacting resilience may differ for neurodivergent populations, especially given that neurodivergent people can face specific adversities related to their neurodivergence. For instance, neurodivergent people can face stressors related to living in a world that is not designed to accommodate them^19^. They may more commonly encounter discrimination, stigma, and victimization^20^, and at a group level, genetic and environmental factors may contribute to increased risk for somatic and psychiatric disorders^21^. As resilience offers a lens for understanding heterogeneity in outcomes observed in neurodivergent populations, while also presenting new avenues for support, exploration of resilience factors in neurodivergence is required.

Given the bio-psycho-social nature of factors influencing resilience, the bio-psycho-social framework and classification system offered by the World Health Organization’s (WHO) International Classification of Functioning (ICF)^22^ is appropriate for investigating the factors underpinning resilience in neurodivergent populations. The ICF is a framework and classification system that focuses on the concepts of functioning, referring to the interaction between an individual, their activities, participation, and surrounding contexts. Unlike biomedical and deficit-focused approaches to understanding disability, the ICF is diagnostically neutral, capable of capturing both individual strengths and difficulties as well as environmental barriers and facilitators, and conceptualizes functioning and disability as arising from an interaction between an individual and their contexts^22^. Its classification system comprises nearly 1700 hierarchically organized codes covering body functions, body structures, activity and participation, and environmental factors. The ICF is endorsed by 194 WHO Member States and is intended to provide a common language for use across various stakeholders and settings, including research, clinical practice, and policy. Due to the comprehensive nature of the ICF, the WHO endorses the development of Core Sets or short lists of codes specific to particular contexts and populations, facilitating their practical implementation and providing minimum standards for assessment and reporting^23^.

This study is part of a larger series of work using the ICF to explore resilience in neurodivergence^24^. This larger series of work aims to identify the bio-psycho-social factors that may be important for risk and resilience in neurodivergence and to develop ICF Core Sets for resilience in neurodivergence^24^. The development of such ICF Core Sets involves a rigorous process outlined by the WHO and ICF Research Branch, comprising several studies aiming to gain broad and diverse perspectives on factors relevant to resilience in neurodivergence. We previously used the ICF to explore resilience factors identified in the extant scientific literature^14^. Here, we add to this literature by presenting an investigation of international health professionals’ perspectives on risk and resilience in neurodivergence using the ICF to identify risk and resilience factors. Further, given that the operation of resilience factors is influenced by a range of contextual factors and circumstances, we also sought to explore whether professionals perceive risk and resilience factors to differ for neurodivergent populations compared to other populations and whether factors differ based on neurodivergent conditions, gender of neurodivergent individuals, or country income. Such an in-depth bio-psycho-social investigation, drawing on the perspectives of professionals and utilising the standard system of the ICF, has not been performed previously and may offer unique insights into resilience in neurodivergence.

## Method

### Design

This work is undertaken within the context of Risk and Resilience in Developmental Diversity and Mental Health (R2D2-MH; https://www.r2d2-mh.eu/), a European Union Horizon project. The current study forms part of a larger project aiming to develop WHO ICF Core Sets for risk and resilience in neurodivergence^24^. An international online survey following ICF Research Branch methodology^23^ was employed to identify factors important for risk and resilience in neurodivergence from the perspectives of clinicians and professionals. The authorship team comprised both neurotypical and neurodivergent authors. This was a primarily exploratory study and, therefore, not guided by specific hypotheses.

## Participants

The sample included clinicians and professionals with experience in the area of neurodivergence or working with neurodivergent individuals. Participants were eligible if they had: (1) a background in one or more of the following areas: psychology, psychiatry, occupational therapy, speech and language pathology, physiotherapy, neurology, social work, or other relevant backgrounds, and (2) over 5 years of practical experience in the area of neurodivergence.

In the context of this work, neurodivergence is defined as individuals with divergent neurological functioning owing to differences in early neurological development and maturation. This includes those with inborn or child-onset conditions, inclusive of neurodevelopmental conditions, genetic syndromes, prematurity, or other conditions arising in early development. These conditions are all neurodevelopmental in nature and have lifelong impacts on functioning. This broad definition is employed due to the considerable overlap between these conditions and their common co-occurrence^25,26^, and to enable continuity with previous works conducted as part of the broader project^14,24^.

An internationally diverse sample was sought, aiming to capture representation from the six WHO regions (Africa, the Americas, Southeast Asia, European, Eastern Mediterranean, and Western Pacific) as per WHO and ICF Research branch guidelines^23^. Sampling was purposive and potential participants were identified via professional networks, organizations, editorial boards for key journals in the field, clinical and research centers, universities, and publications. A snowball sampling approach was also used wherein participants could identify other individuals meeting the inclusion criteria.

A total of 784 professionals were identified and contacted with information about the study. Of those individuals, 13 declined, primarily because they believed that they held insufficient expertise, and 561 did not respond, even after three reminders. A total of 210 individuals accessed the survey, with a further 12 excluded due to ineligibility, resulting in 198 respondents completing and being included in the survey. Participants represented all six WHO regions (38 different countries) and a variety of professional backgrounds and demographics (Table 1).

**Table 1:** Socio-demographic information of professional respondents (*N* = 198).

	*N* (%)
**Demographics of professionals**	
Background*	
Psychology	45 (23%)
Occupational Therapy	34 (17%)
Physician/Psychiatry	29 (15%)
Speech and Language Pathology	29 (15%)
Special Education	15 (18%)
Physiotherapy	12 (6%)
Psychotherapy	12 (6%)
Coaching	9 (5%)
Social Work/Counselling	9 (5%)
Nursing	8 (4%)
Other	17 (8.6%)
**WHO Region**	
European region	75 (38%)
Western Pacific Region	41 (21%)
Region of Americas	41 (21%)
African Region	19 (10%)
Southeast Asian Region	12 (6%
Eastern Mediterranean Region	10 (5%)
Main duties performed *	
Clinical	134 (67.7%)
Research	119 (60.1%)
Teaching	105 (53%)
Management	54 (27.3%)
Other	12 (6.1%)
**Sex**	
Female	134 (68%)
Male	63 (32%9
Missing	1 (1%)
**Demographics of groups with which professionals hold experience**	
Age groups*	
Infants (0–5 years)	110 (55.6%)
Children (6–12 years)	140 (70.7%)
Youth (13–18 years)	134 (67.7%)
Young adults (19–25 years)	101 (51%)
Adults (26 + years)	77 (38.9%)
Diagnostic groups*	
Autism	174 (87.9%)
ADHD	151 (76.3%)
Intellectual disability/Global developmental delay	129 (65.2%)
Communication disorders	106(53.5%)
Specific learning disorders	76 (38.4%)
Genetic syndromes	75 (37.9%)
Prematurity	65 (32.8%)
Motor Disorders	62 (31.3%)
Other	18 (9.1%)
Socio-economic groups served*	
Low socio-economic status	118 (59.6%)
Middle socio-economic status	166 (83.8%)
High socio-economic status	86 (43.4%)
Don’t know	8 (4%)
Don’t want to state	5 (2.5%)

*Indicates items where respondents could select multiple options.

## Survey

The survey design was informed by guidelines provided by the WHO and ICF Research branch for the development of ICF Core Sets^23^. It comprised three parts. Part one included questions related to demographics of the professionals and the characteristics of the neurodivergent populations with which they held experience, while parts two and three contained open-ended questions about the factors that participants believed were important for risk (part two) and resilience (part three) in neurodivergence. Parts two and three were formulated to capture each ICF component (body functions, body structures, activities, participation, environment, and personal factors). For example, “Based on your experiences, what are the physical and mental factors that are important to consider when it comes to resilience?”. Questions related to whether there were risk or resilience factors specific to gender or to neurodivergent individuals compared to neurotypical individuals were also included. Participants were also provided with key definitions to consider when answering the questions.

## Data collection

The online survey was administered via Karolinska Institutet (KI) Survey, a cloud-based service. Potential participants identified via recruitment avenues and/or the snowball method were contacted via email with information about the study and invited to participate with a link to the online survey. Participants were provided with up to three reminders to complete the survey. Participants provided informed consent via the survey platform.

## Identification of meaningful concepts and linking to the ICF(-CY)

Responses were analysed using an established two-phase procedure for the linking of qualitative data to the ICF^27^. In phase one, a deductive qualitative content analysis approach was used to develop meaningful concepts from the responses. Meaningful concepts are units of text extracted based on their meaning in relation to risk or resilience in neurodivergence. (Refer to Supplementary Table 1 for an example of this process). Extracted concepts were then linked to the ICF Child Youth (CY) version^28^. The ICF(-CY) was selected as this represents the most comprehensive form of the ICF, containing codes relevant to developing individuals^28^, and has been used previously for similar Core-Set developments in neurodevelopmental conditions^29,30^. In addition, although the ICF(-CY) does not classify personal factors (e.g., factors such as age, gender, lifestyle, and outlook, which can influence functioning), we deemed that these factors may play a role in the risk or resilience of neurodivergent individuals, so we also linked concepts, where appropriate to the personal factor classification system outlined by Grotkamp et al.^31^. Linking is a process of applying relevant ICF-CY (or personal factor) codes to the meaningful concepts. All linking was performed according to linking rules outlined by WHO^27^. As per linking rules, where possible, concepts were linked to the most precise ICF(-CY) or personal factor code. Where they could not be linked, meaningful concepts were coded as “not definable (ND)” where there was insufficient information to make a decision, “not covered (NC)” was applied when the concept was not covered by the ICF or personal factor classification system, while “health condition (HC)” was applied when the concept referred to a specific diagnosis or health condition. ICF linking was performed by one researcher, with 10% of concepts also linked by a second researcher to enhance the rigour of the linking process. This percentage was chosen as a reasonable balance between ensuring consistency and maintaining practical feasibility. If inter-rater agreement was found to be low, an additional 10% of concepts would be linked to further assess and improve reliability. Both researchers had undergone training on ICF linking from the ICF Research branch and have extensive experience with the ICF and ICF linking. Inter-rater agreement was *k* = 0.72 (CIs: *k =* 0.69–0.75), indicating substantial agreement. Disagreements were resolved via discussion, and the linking for the remainder of the concepts were refined. Examples of the linking process is contained in the supplement (Supplementary Table 1).

### Data analysis

Data were analysed separately for risk and resilience responses. Observation of several responses and meaningful concepts in response to survey questions related to risk were found to be more appropriately resilience factors (for example, where responses were worded in the positive or it was indicated in the response that factors mitigated risk). Thus, prior to further analysis and presentation of results, those meaningful concepts and corresponding ICF linking that were deemed to be more appropriately coded as resilience factors were integrated into the data relating to resilience. Frequency analysis was performed on codes related to risk and resilience separately to identify ICF codes specifically related to risk and resilience. ICF codes were linked to the most precise level possible, but for the purposes of reporting, all codes were converted to the second-level category (e.g., b1521 – emotion regulation becomes b152 emotion). Where responses from an individual respondent were linked to the same second-level category, they were counted only once. We present both the absolute number of responses in which the ICF code was identified as well as the relative percentage. In addition, we further examined differences in the distribution of ICF codes across neurodivergent populations and by country income level. Two additional qualitative questions were also analysed: one exploring whether professionals perceived differences in risk and resilience factors across genders, and another examining perceived differences based on neurodivergence (compared to neurotypicality). The latter was linked to the ICF using the outlined comparisons in the distribution of ICF codes between neurodivergent and neurotypical individuals. Exploration of the gender-related question revealed that many concepts identified by the professionals could not be adequately captured through ICF linking, thus to preserve nuance, a content analysis approach was adopted.

### Ethical considerations

The research was conducted in accordance with the Declaration of Helsinki^32^. Ethical approval was obtained from the Swedish Ethical Review Authority (2023-00127-01). Participants provided informed consent via the survey platform prior to participating. Upon completion of the survey, participants were provided with a knowledge pack containing information about the ICF and the option to receive an online gift voucher valued at SEK 200 (18 EUR).

## Results

A total of 7749 meaningful concepts were extracted from the responses of 198 professionals. These concepts were linked to a total of 7276 ICF(-CY) and personal factor codes, of which 284 codes were unique. Meaningful concepts were also linked to many ND (k = 647), NC (k = 181), and HC codes (k = 155).

### Risk

A total of 3102 meaningful concepts related to risk were identified and linked to 2895 ICF(-CY) and personal factor codes, of which 240 were unique. NC (k = 225), ND (k = 297), and HC (k = 130) codes were also applied. Of the 240 unique ICF(-CY) and personal factor codes, 77 appeared in at least 5% of the responses (range 5–62%). Each of the five components (body functions, body structures, activities and participation, environmental and personal factors) are included. Environmental factors were the most commonly linked component (25%), followed by activities and participation (23%), body functions (21%), body structures (17%) and personal factors (14%). Table 2 presents the absolute and relative frequencies of the second-level ICF codes related to risk across the five components.

**Table 2:** Absolute (f) and relative (%) frequencies of ICF (-CY) and personal factor codes linked to risk. Organized in descending order within each ICF(-CY) component.

Second-level ICF(-CY) or personal factor code	f	%
**Body functions**		
b152 Emotional functions	61	31%
b126 Temperament and personality functions	49	25%
b156 Perceptual functions	48	24%
b299 Sensory functions and pain, unspecified	45	23%
b117 Intellectual functions	38	19%
b760 Control of voluntary movement functions	28	14%
b164 Higher-level cognitive functions	27	14%
b189 Specific mental functions, other specified and unspecified	24	12%
b130 Energy and drive functions	21	11%
b125 Dispositions and intra-personal functions	20	10%
b167 Mental functions of language	15	8%
b230 Hearing functions	13	7%
b122 Global psychosocial functions	12	6%
b134 Sleep functions	12	6%
b160 Thought functions	10	5%
b210 Seeing functions	10	5%
**Body structures**		
s110 Structure of brain	123	62%
s730 Structure of upper extremity	18	9%
s770 Additional musculoskeletal structures related to movement	16	8%
s199 Structure of the nervous system, unspecified	15	8%
s210 Structure of eye socket	14	7%
s220 Structure of eyeball	14	7%
s250 Structure of middle ear	13	7%
s260 Structure of inner ear	13	7%
s750 Structure of lower extremity	13	7%
s320 Structure of mouth	11	6%
s410 Structure of cardiovascular system	11	6%
s240 Structure of external ear	10	5%
s299 Eye, ear and related structures, unspecified	9	5%
**Activity and participation**		
d399 Communication, unspecified	47	24%
d920 Recreation and leisure	37	19%
d820 School education	33	17%
d760 Family relationships	29	15%
d570 Looking after one’s health	26	13%
d729 General interpersonal interactions, other specified and unspecified	26	13%
d850 Remunerative employment	24	12%
d750 Informal social relationships	18	9%
d159 Basic learning, other specified and unspecified	17	9%
d240 Handling stress and other psychological demands	14	7%
d839 Education, other specified and unspecified	14	7%
d910 Community life	14	7%
d720 Complex interpersonal interactions	13	7%
d250 Managing one’s own behaviour	11	6%
d330 Speaking	11	6%
d199 Learning and applying knowledge, unspecified	10	5%
d499 Mobility, unspecified	9	5%
d599 Self-care, unspecified	9	5%
**Environmental factors**		
e310 Immediate family	54	27%
e585 Education and training services, systems and policies	44	22%
e399 Support and relationships, unspecified	43	22%
e165 Assets	36	18%
e580 Health services, systems and policies	26	13%
e599 Services, systems and policies, unspecified	26	13%
e460 Societal attitudes	25	13%
e325 Acquaintances, peers colleagues, neighbours and community members	24	12%
e320 Friends	22	11%
e499 Attitudes, unspecified	18	9%
e298 Natural environment and human-made changes to environment, other specified	14	7%
e410 Individual attitudes of immediate family members	14	7%
e425 Individual attitudes of acquaintances, peers colleagues, neighbours and community members	13	7%
e110 Products or substances for personal consumption	12	6%
e155 Design, construction and building products and technology of buildings for private use	12	6%
e150 Design, construction and building products and technology of buildings for public use	11	6%
e360 Other professionals	11	6%
e455 Individual attitudes of other professionals	9	5%
e570 Social security services, systems and policies	9	5%
**Personal factors**		
i525 Financial status	82	41%
i530 Societal status	64	32%
i515 Residential status	42	21%
i535 Cultural status	34	17%
i110 Chronological age	28	14%
i122 Social sex (gender)	26	13%
i540 Ethnic affilitation	18	9%
i510 Family status	15	8%
i120 Biological sex	13	7%
i410 World view	13	7%
i550 Educational status	9	5%

Codes in the body functions component captured mental functions (b1), sensory functions and pain (b2), and neuromuscular and movement and related functions (b7), representing three of the eight chapters. Mental functions (b1) were the most represented chapter, with 12 out of the 15 second-level codes appearing in this component falling into this chapter. Emotion functions (b152) was the most frequently linked code, which in most cases referred to difficulties regulating emotions. Temperament and personality functions (b126), which tended to refer to tendencies such as being introverted, pessimistic, or less curious or open to experiences, and intellectual functions (b117) were also commonly identified risk factors in the mental functions domain. Perceptual functions (b156) and sensory functions, including hearing (b230) and seeing (b210), were most often related to difficulties associated with hyper-sensitivity but also hyposensitivity. The sole code representing chapter b7 was control of voluntary movement functions (b760), which referred to coordination difficulties and difficulties in fine and gross motor skills.

Responses covered five of the eight body structure components: structure of the nervous system (s1), the eye, ear, and related structures (s2), structures involved in voice and speech (s3), structures of the cardiovascular, immunological, and respiratory systems (s4) and structures related to movement (s7). Structure of the brain (s110) was the most frequently linked second-level code in this domain.

Activities and participation codes captured eight out of the nine chapters in this component, including learning and applying knowledge (d1), general tasks and demands (d2), communication (d3), mobility (d4), self-care (d5), interpersonal interactions and relationships (d7), major life areas (d8) and community, social and civic life (d9). Here, communication, unspecified, was the most frequently linked code, referring to a range of difficulties associated with communication. Difficulties with speaking specifically was linked to 6% of responses. Many codes, such as school education (d820), remunerative employment (d850), recreation and leisure (d920), and community life (d910), tended to refer to difficulties or lack of engagement in these life areas as risks. Codes relating to interpersonal interactions and relationships (d7) tended to capture difficulties engaging in these relationships.

All five chapters of the environmental factors component were covered, including products and technology (e1), natural environment and human-made changes to the environment (e2), support and relationships (e3), attitudes (e4), and services, systems, and policies (e5). Supports and relationships (e3), in most cases, referred to poor or non-existent support, such as family or friends, while attitudes (e4) referred to various attitudes and beliefs held by those close to the neurodivergent person or in society. Regarding personal factors, most codes related to life situations and socio-economic/cultural factors (i5) were covered, but other chapters, including general personal characteristics (i1), and attitudes, action-related skills, and behavior patterns (i4), were also covered.

Although HC codes are not typically examined in the context of studies using the ICF, we decided to summarize these codes, given that they were frequently raised as risk factors for neurodivergent individuals by respondents (k = 130). When examining these HC codes, we identified that the presence of psychiatric or mental health conditions was commonly identified as a risk factor (k = 43, 33%). When they were explicitly identified by respondents, these commonly included anxiety (k = 20, 15%), depression (k = 11, 8%), and personality disorders (k = 8, 6%).

Having more than one neurodevelopmental diagnosis was also commonly a risk factor identified by respondents (k = 29; 22%). Within these responses, some simply highlighted “dual” or “other” neurodevelopmental conditions, while others raised specific conditions, most commonly epilepsy (k = 8, 6%) and communication/language conditions (k = 4, 3%). Co-occurring physical or somatic (k = 21, 16%), and more generally co-occurring conditions (k = 14, 11%) were also identified as risk factors.

### Resilience

A total of 4647 meaningful concepts related to resilience were extracted and linked to 4381 ICF(-CY) and personal factor codes, of which 245 were unique. NC (k = 239), ND (k = 394), and HC (k = 25) codes were also applied. Of the 244 unique codes identified, 80 appeared in 5% or more of the responses (5% − 65%). All five of the ICF components were represented. Environmental factors (35%) and Activity and participation (29%) were most frequently linked in relation to resilience. Body functions (18%), personal factors (10%), and body structures (9%) were also linked. Table 3 presents the absolute and relative frequencies of the second-level ICF codes relating to resilience across the five components.

**Table 3:** Absolute (f) and relative (%) frequencies of ICF (-CY) and personal factor codes linked to resilience. Organized in descending order within each ICF(-CY) component.

Second-level ICF(-CY) or personal factor code	f	%
**Body functions**		
b126 Temperament and personality functions	105	53%
b152 Emotional functions	74	37%
b164 Higher-level cognitive functions	72	36%
b125 Dispositions and intra-personal functions	54	27%
b117 Intellectual functions	48	24%
b134 Sleep functions	31	16%
b130 Energy and drive functions	24	12%
b156 Perceptual functions	23	12%
b299 Sensory functions and pain, unspecified	23	12%
b167 Mental functions of language	15	8%
b760 Control of voluntary movement functions	13	7%
b122 Global psychosocial functions	12	6%
b160 Thought functions	12	6%
b189 Specific mental functions, other specified and unspecified	11	6%
**Body structures**		
s110 Structure of brain	129	65%
s198 Structure of the nervous system, other specified	18	9%
s770 Additional musculoskeletal structures related to movement	17	9%
s410 Structure of cardiovascular system	16	8%
s199 Structure of the nervous system, unspecified	13	7%
s420 Structure of immune system	12	6%
s730 Structure of upper extremity	9	5%
**Activity and participation**		
d920 Recreation and leisure	100	51%
d750 Informal social relationships	58	29%
d820 School education	52	26%
d760 Family relationships	50	25%
d570 Looking after one’s health	49	25%
d910 Community life	49	25%
d850 Remunerative employment	46	23%
d399 Communication, unspecified	45	23%
d240 Handling stress and other psychological demands	40	20%
d720 Complex interpersonal interactions	31	16%
d729 General interpersonal interactions, other specified and unspecified	30	15%
d839 Education, other specified and unspecified	30	15%
d159 Basic learning, other specified and unspecified	28	14%
d799 Interpersonal interactions and relationships, unspecified	25	13%
d250 Managing one’s own behaviour	19	10%
d499 Mobility, unspecified	18	9%
d599 Self-care, unspecified	14	7%
d855 Non-remunerative employment	14	7%
d230 Carrying out daily routine	13	7%
d815 Preschool education	13	7%
d830 Higher education	12	6%
d930 Religion and spirituality	11	6%
d177 Making decisions	10	5%
**Environmental factors**		
e310 Immediate family	92	46%
e399 Support and relationships, unspecified	85	43%
e585 Education and training services, systems and policies	51	26%
e580 Health services, systems and policies	49	25%
e320 Friends	43	22%
e499 Attitudes, unspecified	32	16%
e325 Acquaintances, peers colleagues, neighbours and community members	31	16%
e599 Services, systems and policies, unspecified	30	15%
e410 Individual attitudes of immediate family members	27	14%
e165 Assets	26	13%
e460 Societal attitudes	25	13%
e155 Design, construction and building products and technology of buildings for private use	20	10%
e360 Other professionals	20	10%
e298 Natural environment and human-made changes to environment, other specified	18	9%
e110 Products or substances for personal consumption	17	9%
e425 Individual attitudes of acquaintances, peers colleagues, neighbours and community members	17	9%
e525 Housing services, systems and policies	16	8%
e570 Social security services, systems and policies	16	8%
e199 Products and technology, unspecified	15	8%
e150 Design, construction and building products and technology of buildings for public use	14	7%
e590 Labour and employment services, systems and policies	14	7%
e198 Products and technology, other specified	13	7%
e330 People in positions of authority	12	6%
e530 Utilities services, systems and policies	12	6%
e555 Associations and organizational services, systems and policies	12	6%
e398 Support and relationships, other specified	10	5%
e575 General social support services, systems and policies	10	5%
e465 Social norms, practices and ideologies	9	5%
**Personal factors**		
i515 Residential status	58	29%
i525 Financial status	50	25%
i436 Empowerment	31	16%
i530 Societal status	31	16%
i410 World view	28	14%
i411 Attitude towards one’s own self	22	11%
i535 Cultural status	22	11%
i110 Chronological age	21	11%

Captured body functions relating to resilience were similar to those captured for risk, with captured chapters including mental functions (b1), sensory functions and pain (b2), and neuromuscular and movement and related functions (b7). Temperament and personality functions (b126) was the most frequently linked code, which most often referred to extroversion, optimism, and openness to experience. Dispositions and intrapersonal functions (b125) capturing aspects such as approachability and persistence were also commonly linked. Over a third of respondents (37%) were linked to emotion functions (b152) which in the majority of cases captured emotion regulation abilities as important for resilience. Intellectual functions (b117) and higher-level cognitive functions, which in relation to resilience referred to abilities in problem-solving, insight, cognitive flexibility, and organization and planning, were also commonly identified. Sleep functions (b134), capturing the amount and quality of sleep, energy and drive functions (b130), perceptual functions (b156), and sensory functions and pain were commonly identified as resilience factors. Three of the eight body structure components were covered, including structure of the nervous system (s1), structures of the cardiovascular, immunological, and respiratory systems (s4) and structures related to movement (s7). Similar to responses for risk, structure of the nervous system (s1) was the most linked chapter in this component.

Eight out of the nine chapters of the activity and participation component were captured, including learning and applying knowledge (d1), general tasks and demands (d2), communication (d3), mobility (d4), self-care (d5), interpersonal interactions and relationships (d7), major life areas (d8) and community, social and civic life (d9). Linking this chapter referred to engagement in areas such as education, esmployment, leisure, and community life as important for resilience. Different forms of relationships, most often informal relationships such as friendships and family relationships, were identified commonly by respondents as resilience factors. Other commonly occurring codes related to communication, and abilities to handle stress or other demands.

All five chapters of the environmental factors component were covered. Codes in this component most often refer to the presence of positive support networks such as family, friends, and other community members. Acceptance, understanding, and positive attitudes from these individuals, as well as on a broader societal level, were also identified as resilience factors. Many codes are also related to services and systems, referring to access to quality education, health, and other services. Three out of five personal factor chapters were covered in relation to resilience, including general personal characteristics (i1), attitudes, action-related skills, behavior patterns (i4), and life situations and socioeconomic/cultural factors (i5). Similar to codes for risk, many responses referred to an individual’s socioeconomic and residential status, with living in higher-income countries and having a higher socioeconomic status found to be a resilience factor.

### Differences in risk and resilience factors across neurodivergence

**Fig. 1:**
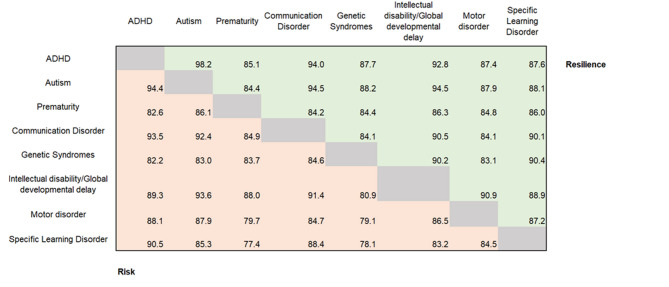
Percentage (%) of codes shared between conditions. Bottom (red) indicates shared codes for risk and top (green) indicates shared codes for resilience.

Once we had established risk and resilience factors across the entire sample, we further explored whether there were specific risk or resilience factors more or less salient for particular types of neurodivergence. When examining risk and resilience codes appearing in 5% or more of the responses, there were very few differences between conditions. A total of 44 codes for risk and 63 codes for resilience were shared across all conditions (Supplementary Table 2). When examining the percentage of codes shared between conditions, there was a high degree of overlap, ranging from 77.4% to 94.4% of shared codes for risk and 83.1% to 98.1% for resilience (Fig. 1).

Fourteen risk and 15 resilience codes appeared uniquely in relation to one condition. These unique factors all appeared in very low frequencies (codes appearing in ~ < 6% of responses within specific conditions), suggesting that differences may be attributable to noise within the sample. For example, eating (d550) was a risk factor uniquely attributed to intellectual disability/global developmental delay, appearing in 5% (*n* = 7 out of 129 total responses corresponding to this condition) of responses for this condition, while hearing functions (b230) was a resilience factor identified only for prematurity (*n* = 4, 6% out of 65 total responses corresponding to this condition).

### Differences in risk and resilience factors based on gender

Of the 198 respondents, 140 (71% of the sample) thought that there were gender differences when considering risk and resilience in neurodivergent populations. Responses highlighted a range of factors that may function differently across gendets but, in most cases, referred to risk factors. An initial examination of the responses indicated that many would not be linkable to the ICF or personal factor codes; therefore, we proceeded with a content analysis approach.

In most cases, respondents appeared to indicate that being a female was associated with more risk than compared to males. Specific risk and resilience factors for females and males provided by the respondents are provided in Supplementary Tables 3 and 4, respectively. It should also be noted that several responses (k = 11) reported that being in a minority gender (e.g., non-binary) or not identifying with birth sex was also a risk factor. Few responses provided reasoning, but one suggested that this may be due to increased discrimination.

Risk factors for both males and females included social roles and expectations. For females, social expectations and roles were reported to place additional demands or burdens on individuals, such as requirements to undertake caretaking roles or household management and to appear social. Some respondents indicated that demands to undertake particular caretaking roles may be more prevalent in non-Western countries. For males, some respondents noted that social norm expectations for males to financially provide, not express emotion, and not seek help were risk factors.

Other risk and resilience factors appeared to be more specific to females and males. For instance, later diagnosis or missed diagnosis and camouflaging or masking were also highlighted as risk factors for females, whereas only one response highlighted camouflaging as a risk factor for males specifically. In some cases, camouflaging was also reported to be a resilience factor for females, enabling them to mask certain traits that may be considered adaptive in certain circumstances. A number of responses also highlighted that females may be more likely to experience sexual abuse or other forms of abuse or discrimination as risks. In contrast, exposure to violence, drugs, and criminality was identified as a risk factor for males. Internalizing symptoms and self-injury were risk factors identified for females, while externalizing behaviors such as aggression or other challenging behavior were risk factors for males. In general, females were reported to be more socially inclined and have greater social skills, providing a greater social support network, which was a resilience factor.

### Differences in risk and resilience factors based on country income

To examine whether identified risk and resilience factors differed across different country income levels, we divided respondent countries into income categories using the World Bank Group assignment for world economies^33^. Countries were allocated to either low, lower-middle, upper-middle, or high-income groups. In our sample, most respondents were from high-income countries (*n* = 151), and fewer were from upper-middle (*n* = 30) and lower-middle-income (*n* = 15) countries. No respondents were from low-income countries. A total of 49 codes for risk and 47 codes for resilience were shared across the three income levels (Supplementary Table 5).

High and upper-middle-income countries had the greatest number of shared codes (76.4% risk and 72.1% resilience), followed by higher-income and lower-middle (72.3% risk and 70.0% resilience) and upper-middle and lower-middle income (68.2% risk and 66.0% resilience). Differences across country income levels appeared mostly in lower frequencies. For example, Social Security services, systems, and policies (e570) was a resilience factor uniquely identified for high-income countries, identified by 15 respondents (10% of high-income respondents), while self-care, unspecified (d599), was a risk factor uniquely identified by respondents from lower-middle-income countries (3 respondents, 20% of lower middle respondents). Again, it is possible that unique factors may be attributable to noise within the sample.

### Differences in risk and resilience factors between neurodivergent and neurotypical individuals

Of the 198 responses,157 (79% of the sample) reported that they thought that there were risk and resilience factors that were different for neurodivergent compared to neurotypical populations. In contrast, 27 (14%) respondents explicitly indicated that they did not believe that risk or resilience factors differed between neurotypes, while six reported that they were unsure (3%). Some respondents (*n* = 11) indicated that they thought that risk and resilience factors were the same, but that the magnitude of the impact may differ for neurodivergent individuals. In the vast majority of cases, respondents identified risk factors that were unique or more salient for neurodivergent individuals rather than resilience factors.

First, we linked responses to the ICF-CY and personal factor codes to explore risk and resilience factors that may differ between neurotypical and neurodivergent individuals. In total 627 meaningful concepts were extracted from responses to this question, which were linked to 506 ICF codes, of which 73 were unique. Concepts were also frequently assigned NC (k = 99), ND (k = 56), and HC (k = 26) codes. Seven unique ICF codes were linked to 5% or more of responses (Supplementary Table 6) Body functions included sensory (b299) and perceptual (b156) functions that captured difficulties and differences in sensory processing and perception that could also act as specific risk factors for neurodivergent individuals. Higher-level cognitive functions (b164) were also identified as a risk factor that was more salient for neurodivergent individuals. However, some respondents also identified this may be a more salient aresilience factor among neurodivergent individuals, who may demonstrate a greater ability to explore different solutions to problems.

Environmental factors, in most cases, referred to attitudes (e460, e499), wherein stigma and a lack of understanding and acceptance were risk factors unique to neurodivergent individuals. Environmental codes also included supports and relationships (e399), which were reported to have a greater impact, both positively and negatively, for neurodivergent individuals. The sole activity and participation code captured difficulties with communication (d399) that could operate as a greater risk factor in neurodivergent populations.

Given the high number of ND, NC, and HC codes, we also examined these concepts to identify other notable risk and resilience factors that may operate uniquely or more saliently among neurodivergent populations not covered by the ICF or personal factor codes. Several responses indicated that neurodivergent individuals may be more prone, or have a greater exposure, to some specific risk factors, such as violence or abuse, compared to neurotypical individuals. Responses also indicated that neurodivergent individuals can experience a greater prevalence of mental health diagnoses, such as anxiety or depression, which can also operate to increase risk. Regarding social attitudes and norms, some respondents (*n* = 4) noted that the need to camouflage or mask behaviors were a risk factor uniquely impacting neurodivergent individuals. On the other hand, special interests and strengths, such as creativity, were identified as more salient resilience factors for neurodivergent individuals.

## Discussion

This study examined professional and clinician perspectives on risk and resilience factors for functioning, well-being, and mental health outcomes in neurodivergence. A range of bio-psycho-social factors influencing both risk and resilience in neurodivergence were identified by the professionals, highlighting the multi-faceted and complex nature of resilience in neurodivergence.

Neurological diversity has primarily been viewed through the lens of the medical model, emphasizing individual-level contributors to risk and resilience. For example, previous research has tended to focus on core individual traits, characteristics, or abilities that place neurodivergent individuals at risk of poor outcomes, while clinical practice has tended to focus interventions on individual abilities, such as emotion regulation^34,35^. Though professionals in our study perceived that individual factors do indeed play a role in both risk and resilience in neurodivergence, their responses indicate that these factors may represent only one component of the factors that may be acting to contribute to risk and resilience in neurodivergence. Though also identifying many individual factors, professionals identified environmental aspects and factors related to activity and participation as more frequently influencing risk and resilience in neurodivergence. These findings align with previous research using the ICF that has also shown the influential role of environments on the functioning of neurodivergent individuals^8,9^, and aligns more broadly with ongoing paradigms shifts in the field that conceptualize disability in neurodivergence as arising from the interaction between an individual and their environments^2,10^. Insights from professionals thus suggest that greater emphasis should be placed on examining the contribution of environments to the outcomes of neurodivergent individuals and exploring person-environment fit.

The bio-psycho-social factors identified by the professionals and clinicians appear to be similar to those identified in other populations. For instance, multi-systematic processes spanning bio-psycho-social domains have also been found to influence resilience in mental health^36^. In these populations, individual factors such as identity and emotion regulation, and environmental factors such as family, parental, and community support are consistently identified in the literature^11,36,37^, mirroring those factors identified in our current study. Thus, our findings seem to suggest the existence of some “shared” or “universal” resilience factors that may also apply to neurodivergent populations. However, in interpreting the apparent similarities between neurodivergent and other populations, it may be important to consider that the operation of these factors may differ. For example, friendships (e320) represent a potential “shared” resilience factor that has been identified in the extant literature^38,39^ and in our current study. Although friendships thus appear important, there may be differences in what is considered desirable and important for friends (quality compared to quantity), and there may be unique challenges experienced by neurodivergent individuals, making it more difficult for friendships to operate as a resilience-inducing factor in these populations^40^. Given the unique experiences of neurodivergent individuals (e.g., the double empathy problem, to name just one example), the exact roles and contributions that identified factors may play in risk and resilience for neurodivergent populations need to be considered. Examining first-person accounts and lived experience perspectives on risk and resilience may therefore be particularly important to lend insights into how the nature of neurodivergence may shape the operation of specific risk and resilience factors.

At the same time, although factors identified by professionals in the current study seem to align with those identified by previous research in other populations^11,36,37^, professionals highlighted several factors that may be unique or more salient for neurodivergent individuals than for neurotypical individuals. Several respondents perceived the underlying factors to be similar between neurotypical and neurodivergent individuals, but the magnitude of their impact was greater for neurodivergent individuals. Such ideas align with theories related to differential sensitivity, which purport that individuals can have varying levels of sensitivity to environmental conditions^41^. Those who are less sensitive to environments are more able to withstand adverse conditions but might also benefit less from optimal environments, while those who are more sensitive might be more greatly impacted by adverse environments but also show more positive outcomes when exposed to optimal conditions^42^. Heightened sensory sensitivity is well-documented in neurodivergent populations, particularly among autistic individuals and those with ADHD^43,44^. These sensory sensitivities have been associated with a range of poorer outcomes, such as in education^45^. This provides evidence that adverse environments might more greatly impact neurodivergent people; however, it is unknown if heightened sensitivity may also confer advantages under optimal conditions, likely because environments are rarely designed for neurodivergent individuals. Such topics may benefit from further investigation and may point to a need for increased efforts to improve environmental conditions.

Professionals’ believed that that there may be gender-differences to consider when exploring risk and resilience in neurodivergence. These findings might reflect the gender-based differences observed within neurodivergent populations, which have highlighted sex/gender differences across phenotypic, neurobiological, genetic, and other domains^46^. Respondents perceived societal expectations to influence risk and resilience differentially across the genders. These findings align with previous research that has suggested that females/women face greater societal demands and expectations to appear social. These societal demands may play a role in camouflaging, which has been suggested to be more prevalent in females/women. Camouflaging has been shown to contribute to poor mental health^47^ and can also contribute to delayed or missed diagnosis, which can hinder access to support^48^. Though not within the scope of this study, some responses highlighted that those who are within a minority gender (for example, non-binary or other) or who do not identify as their birth sex may experience greater risk than others. There is evidence that neurodivergent individuals may be more likely to report different gender identities^49^, and as gender-diverse individuals already face a higher incidence of adversity^50^, there is a need to examine how risk and resilience among individuals who may experience such intersectional identities.

Cultural variation has been suggested to play a role in shaping the protective and promotive factors that operate in resilience^13^. In conducting this study, we sought to capture the potential influence of this cultural variation on risk and resilience by collecting responses from clinicians and professionals across all WHO regions. We examined potential differences across WHO regions by exploring country income, finding only a few differences in the identified factors, suggesting that factors may be largely similar across WHO regions. However, it is important to note that country income was the only comparison that we explored and is, of course, only one source of potential variation. Different variables, such as social and cultural norms and the availability of resources, are likely also important sources of variation that may also influence the operation of risk and resilience factors. For example, some research has found that predictors of quality of life for autistic adults can differ based on socio-cultural context^51^. There were indeed a large number of codes assigned to only a handful of responses that might have represented variability in risk and resilience factors due to cultural or other variability of the respondents. As environmental factors were frequently identified as influential to risk and resilience in neurodivergence, and because cultural and social background can influence the development of other factors such as identity or individual value, the influence of cultural variation on resilience in neurodivergence warrants investigation, and future studies should directly examine cultural and social norms rather than proxying with income.

Overall, our findings have several theoretical and practical implications for understanding risk and resilience in neurodivergence, for future research, and for informing intervention and support. Limited research to date has explored resilience in neurodivergent populations, and those studies that have looked specifically at resilience have tended to do so from the perspective of resilience as a trait or individual characteristic^14^. By examining resilience as a bio-psycho-social process through the lens of the internationally accepted WHO ICF, our study offers insights into the dynamic and complex nature of resilience in neurodivergence.

That resilience factors identified by professionals seemed to be largely shared across diagnoses, genders, and country income, as well as showing similarities with previous investigations in other populations^12^, suggest that transdiagnostic or systemic approaches spanning multiple populations may be beneficial to explore. Such approaches may hold the potential to inform broader community-level initiatives that may act to promote positive outcomes or buffer against negative outcomes, regardless of population. For example, resilience-building strategies might be effectively promoted more systematically across educational, employment, and other life domains. Whole-school interventions may offer an example of such approaches, which have shown some efficacy in improving some outcomes among youth, particularly in reducing risky behaviours^52^. Still, the fact that professionals perceive certain nuances in neurodivergent populations compared to neurotypical populations, as well as some differences based on gender, suggests that there is also a need for considered approaches and investigation that seek to understand and better define these individual-level differences. Person-centred approaches should be used when working with neurodivergent individuals, but our findings point to certain areas that may be particularly important to examine for specific individuals (e.g., the impact of gender norms or camouflaging for women). From an intervention and support perspective, our findings also suggest that while supporting the development of individual skills is important, looking beyond the individual, especially at the support structures surrounding the person, and facilitating engagement in occupation seems essential.

### Limitations

Although capturing diverse perspectives from a range of disciplines and WHO regions, the majority of responses originated from higher-income countries, and comparisons across country income levels were difficult given small sample sizes in upper-middle and lower-middle income countries and an absence of responses in low-income countries. Similarly, the survey was conducted only in English. Resultantly, it is possible that those from other contexts, especially non-Western and lower-income contexts, were not adequately captured and may differ from those captured in the current survey, and global generalisability cannot be assumed. In addition, it is possible that bias may have been introduced through participants aligning with more neurodiversity-affirming frameworks. This may have been particularly influenced by the terminology used in recruitment material and throughout the study.While the survey was designed based on the ICF research guidelines, the topic of risk and resilience for positive life outcomes is broad. Thus, it is unlikely that we were able to capture the full complexity of risk and resilience in neurodivergence. A high degree of overlap across conditions was found, but in interpreting these findings, it should be noted that respondents often held experience across multiple neurodivergent groups, and thus, the high degree of overlap observed might, in part, be a function of the collective groups with which the respondents held expertise. Respondents, however, rarely indicated that there were specific risk and resilience factors for specific conditions in their responses. We did not capture whether the respondents themselves were neurodivergent, and it is possible that respondents were also neurodivergent, and thus may have presented a dual perspective. Relatedly, it is important to note that the purpose of this study was to capture clinician and professional perspectives on risk and resilience factors in neurodivergence and not those of neurodivergent individuals. For this reason, this study will be combined with future qualitative work exploring the perspectives of neurodivergent individuals themselves to provide a holistic and comprehensive perspective on factors influencing risk and resilience in neurodivergence. The findings we present are also based solely on the perspectives of professionals, and thus future research is needed to more specifically examine the exact role of various risk and resilience factors in influencing outcomes for neurodivergent individuals. Due to the nature of the ICF linking process, there were many meaningful concepts that the ICF did not cover, and thus, some perspectives may not have been fully captured. Finally, in conceptualizing adversity within this work, we did not limit our scope to examining specific adversities (such as abuse or bullying) due to the wide variability in adverse experiences and the risk of overlooking experiences that neurodivergent people may perceive as significant. Nonetheless, we acknowledge that particular risk and resilience factors may be associated with more specific adversities, and these warrant targeted investigation in future research.

## Conclusion

This study forms part of a larger body of work seeking to develop ICF core sets for risk and resilience in developmental diversity. Here, we conducted an international exploration of clinician and professional perspectives on the factors they believe are important for risk and resilience in the face of neurodivergence. Professionals identified a range of bio-psycho-social factors influencing resilience, but most frequently identified environmental and activity and participation factors. Insights from professionals indicate a need to consider resilience in neurodivergence from a bio-psycho-social perspective, providing the basis for future assessment and support provision that enables individuals with neurodivergence to achieve positive life outcomes.

## Supplementary Information

Below is the link to the electronic supplementary material.

Supplementary Material 1

## Data Availability

De-identified data and ICF coding is available upon reasonable request from the corresponding author.
