# Supplementary material for: Resilience in neurodivergence: professional perspectives mapped to the World Health Organisations’ International Classification of Functioning
Source: Sci Rep. 2025 Oct 27;15:37360. doi: 10.1038/s41598-025-25079-0 (PMC12559189; doi:10.1038/s41598-025-25079-0)
Supplement: Supplementary file 1 — Supplementary Material 1 [file 41598_2025_25079_MOESM1_ESM.docx]

**Supplementary Table 1**. Example of extraction of meaningful concepts and ICF(-CY) linking from survey responses.

| **Excerpt of survey response** | **Meaningful concepts extracted** | **ICF (-CY) codes assigned.** |
| --- | --- | --- |
| Good intellectual function. Able to regulate their emotions. Executive well-functioning. Healthy motion habits | Good intellectual functioning | b117 - Intellectual functions |
|  | Emotion regulation abilities | b1521 - Emotion regulation |
|  | Executive functioning | b164 - Higher level cognitive functions |
|  | Healthy motion habits | d5701 - Managing diet and fitness |
| Good family relationship and good parent-child interaction.  School, kindergarten and societal acceptance | Good family relationships | d760 - Family relationships |
|  | Good parent-child interactions | d7600 - Parent-child interactions |
|  | School acceptance | e425 - Individual attitudes of acquaintances, peers, colleagues, neighbours and community members |
|  | Kindergarten acceptance | e425 - Individual attitudes of acquaintances, peers, colleagues, neighbours and community members |
|  | Societal acceptance | e460 - Societal attitudes |

**Supplementary Table 2**. ICF(-CY) and Personal Factor codes shared across all neurodivergent conditions. Only codes appearing in >5% of responses are considered.

| **Shared Risk Codes** | **Shared Resilience Codes** |
| --- | --- |
| b125 Dispositions and intra-personal functions | b122 Global psychosocial functions |
| b126 Temperament and personality functions | b125 Dispositions and intra-personal functions |
| b130 Energy and drive functions | b126 Temperament and personality functions |
| b134 Sleep functions | b130 Energy and drive functions |
| b152 Emotional functions | b134 Sleep functions |
| b156 Perceptual functions | b152 Emotional functions |
| b160 Thought functions | b156 Perceptual functions |
| b164 Higher-level cognitive functions | b164 Higher-level cognitive functions |
| b189 Specific mental functions, other specified and unspecified | b167 Mental functions of language |
| b299 Sensory functions and pain, unspecified | b299 Sensory functions and pain, unspecified |
| b760 Control of voluntary movement functions | b760 Control of voluntary movement functions |
| d240 Handling stress and other psychological demands | d159 Basic learning, other specified and unspecified |
| d399 Communication, unspecified | d240 Handling stress and other psychological demands |
| d570 Looking after one’s health | d250 Managing one’s own behaviour |
| d729 General interpersonal interactions, other specified and unspecified | d399 Communication, unspecified |
| d750 Informal social relationships | d570 Looking after one’s health |
| d760 Family relationships | d720 Complex interpersonal interactions |
| d820 School education | d729 General interpersonal interactions, other specified and unspecified |
| d850 Remunerative employment | d750 Informal social relationships |
| d920 Recreation and leisure | d760 Family relationships |
| e165 Assets | d799 Interpersonal interactions and relationships, unspecified |
| e310 Immediate family | d820 School education |
| e320 Friends | d839 Education, other specified and unspecified |
| e325 Acquaintances, peers colleagues, neighbours and community members | d850 Remunerative employment |
| e399 Support and relationships, unspecified | d855 Non-remunerative employment |
| e410 Individual attitudes of immediate family members | d910 Community life |
| e425 Individual attitudes of acquaintances, peers colleagues, neighbours and community members | d920 Recreation and leisure |
| e460 Societal attitudes | e110 Products or substances for personal consumption |
| e499 Attitudes, unspecified | e150 Design, construction and building products and technology of buildings for public use |
| e580 Health services, systems and policies | e155 Design, construction and building products and technology of buildings for private use |
| e585 Education and training services, systems and policies | e165 Assets |
| e599 Services, systems and policies, unspecified | e198 Products and technology, other specified |
| i110 Chronological age | e199 Products and technology, unspecified |
| i122 Social sex (gender) | e298 Natural environment and human-made changes to environment, other specified |
| i510 Family status | e310 Immediate family |
| i515 Residential status | e320 Friends |
| i525 Financial status | e325 Acquaintances, peers colleagues, neighbours and community members |
| i530 Societal status | e330 People in positions of authority |
| i535 Cultural status | e360 Other professionals |
| i540 Ethnic affiliation | e399 Support and relationships, unspecified |
| s110 Structure of brain | e410 Individual attitudes of immediate family members |
| s199 Structure of the nervous system, unspecified | e425 Individual attitudes of acquaintances, peers colleagues, neighbours and community members |
| s410 Structure of cardiovascular system | e460 Societal attitudes |
| s730 Structure of upper extremity | e499 Attitudes, unspecified |
|  | e530 Utilities services, systems and policies |
|  | e570 Social security services, systems and policies |
|  | e580 Health services, systems and policies |
|  | e585 Education and training services, systems and policies |
|  | e590 Labour and employment services, systems and policies |
|  | e599 Services, systems and policies, unspecified |
|  | i110 Chronological age |
|  | i410 World view |
|  | i411 Attitude towards one's own self |
|  | i436 Empowerment |
|  | i515 Residential status |
|  | i525 Financial status |
|  | i530 Societal status |
|  | i535 Cultural status |
|  | s110 Structure of brain |
|  | s198 Structure of the nervous system, other specified |
|  | s199 Structure of the nervous system, unspecified |
|  | s410 Structure of cardiovascular system |
|  | s420 Structure of immune system |

**Supplementary Table 3.** Risk and resilience factors identified by respondents for females. N represents number of respondents identifying category.

| **Risk or resilience factor** | **Risk (N)** | **Resilience (N)** |
| --- | --- | --- |
| Social roles and expectations | 22 | 0 |
| Missed or later diagnosis | 20 | 0 |
| Camouflaging/masking | 19 | 3 |
| Sexual abuse | 13 | 0 |
| Abuse | 11 | 0 |
| Less likely to receive support | 11 | 0 |
| Internalizing behaviour and symptoms | 7 | 0 |
| Less understanding from others | 5 | 0 |
| Discrimination | 4 | 0 |
| Self-injury | 4 | 0 |
| Greater symptoms when diagnosed | 3 | 0 |
| More emotional/greater emotional Vulnerability | 3 | 0 |
| Social stress | 2 | 0 |
| Physical appearance | 2 | 1 |
| More social support | 0 | 11 |
| Social skills | 0 | 4 |
| Emotional expression | 0 | 3 |
| Sex chromosome buffers | 0 | 2 |
| Emotional regulation | 0 | 2 |

**Supplementary Table 4.** Risk and resilience factors identified by respondents for males. N represents number of respondents identifying category.

| **Risk or resilience factor** | **Risk (N)** | **Resilience (N)** |
| --- | --- | --- |
| Aggression | 7 | 0 |
| Social roles and expectations | 8 | 0 |
| More challenging behaviour | 2 | 0 |
| Drug exposure | 2 | 0 |
| Less family support | 2 | 0 |
| Exposure to criminality | 2 | 0 |
| Not managing school | 2 | 0 |
| Skills (athletic ability/technology) | 0 | 2 |

**Supplementary Table 5. ICF(-CY) and Personal Factor codes shared across all country income levels. Only codes appearing in >5% of responses are considered.**

| **Shared risk** | **Shared resilience** |
| --- | --- |
| b122 Global psychosocial functions | b125 Dispositions and intra-personal functions |
| b125 Dispositions and intra-personal functions | b126 Temperament and personality functions |
| b126 Temperament and personality functions | b130 Energy and drive functions |
| b152 Emotional functions | b134 Sleep functions |
| b156 Perceptual functions | b152 Emotional functions |
| b164 Higher-level cognitive functions | b156 Perceptual functions |
| b189 Specific mental functions, other specified and unspecified | b164 Higher-level cognitive functions |
| b299 Sensory functions and pain, unspecified | b299 Sensory functions and pain, unspecified |
| b760 Control of voluntary movement functions | d159 Basic learning, other specified and unspecified |
| d159 Basic learning, other specified and unspecified | d240 Handling stress and other psychological demands |
| d399 Communication, unspecified | d250 Managing one’s own behaviour |
| d570 Looking after one’s health | d399 Communication, unspecified |
| d720 Complex interpersonal interactions | d570 Looking after one’s health |
| d729 General interpersonal interactions, other specified and unspecified | d729 General interpersonal interactions, other specified and unspecified |
| d750 Informal social relationships | d750 Informal social relationships |
| d760 Family relationships | d760 Family relationships |
| d820 School education | d799 Interpersonal interactions and relationships, unspecified |
| d850 Remunerative employment | d820 School education |
| d910 Community life | d839 Education, other specified and unspecified |
| d920 Recreation and leisure | d850 Remunerative employment |
| e165 Assets | d855 Non-remunerative employment |
| e310 Immediate family | d910 Community life |
| e320 Friends | d920 Recreation and leisure |
| e325 Acquaintances, peers colleagues, neighbours and community members | e150 Design, construction and building products and technology of buildings for public use |
| e399 Support and relationships, unspecified | e155 Design, construction and building products and technology of buildings for private use |
| e410 Individual attitudes of immediate family members | e165 Assets |
| e425 Individual attitudes of acquaintances, peers colleagues, neighbours and community members | e199 Products and technology, unspecified |
| e460 Societal attitudes | e298 Natural environment and human-made changes to environment, other specified |
| e499 Attitudes, unspecified | e310 Immediate family |
| e580 Health services, systems and policies | e320 Friends |
| e585 Education and training services, systems and policies | e325 Acquaintances, peers colleagues, neighbours and community members |
| i110 Chronological age | e399 Support and relationships, unspecified |
| i122 Social sex (gender) | e460 Societal attitudes |
| i510 Family status | e499 Attitudes, unspecified |
| i515 Residential status | e525 Housing services, systems and policies |
| i525 Financial status | e580 Health services, systems and policies |
| i530 Societal status | e585 Education and training services, systems and policies |
| i535 Cultural status | i110 Chronological age |
| i540 Ethnic affiliation | i411 Attitude towards one's own self |
| s110 Structure of brain | i436 Empowerment |
| s199 Structure of the nervous system, unspecified | i515 Residential status |
| s210 Structure of eye socket | i525 Financial status |
| s220 Structure of eyeball | i530 Societal status |
| s250 Structure of middle ear | i535 Cultural status |
| s260 Structure of inner ear | s110 Structure of brain |
| s320 Structure of mouth | s198 Structure of the nervous system, other specified |
| s730 Structure of upper extremity | s770 Additional musculoskeletal structures related to movement |
| s750 Structure of lower extremity |  |
| s770 Additional musculoskeletal structures related to movement |  |

**Supplementary Table 6.** Absolute (f) and relative (%) frequencies of ICF (-CY) codes identified by respondents as being different between neurodivergent and neurotypical individuals.

| **ICF-CY code** | ***f*** | **%/157** |
| --- | --- | --- |
| b156 Perceptual functions | 17 | 11% |
| b299 Sensory functions and pain, unspecified | 16 | 10% |
| e399 Support and relationships, unspecified | 15 | 10% |
| e460 Societal attitudes | 13 | 8% |
| e499 Attitudes, unspecified | 11 | 7% |
| b164 Higher-level cognitive functions | 9 | 6% |
| d399 Communication, unspecified | 9 | 6% |
